# Receiving a diagnosis of young onset dementia: Evidence-based statements to inform best practice

**DOI:** 10.1177/1471301220969269

**Published:** 2020-10-30

**Authors:** Mary O’Malley, Jacqueline Parkes, Jackie Campbell, Vasileios Stamou, Jenny LaFontaine, Jan R Oyebode, Janet Carter

**Affiliations:** UoN Dementia Research & Innovation Centre, Faculty of Health, Education and Society, 6087 University of Northampton, Northampton, UK; Faculty of Health, Education and Society, 6087 University of Northampton, Northampton, UK; Centre for Applied Dementia Studies, Faculty of Health Studies, 1905 University of Bradford, Bradford, UK; Faculty of Brain Sciences, Division of Psychiatry, Maple House, 4919 University College London, London, UK

**Keywords:** young onset dementia, dementia assessment, diagnosis, lived experiences, Delphi methods

## Abstract

**Introduction:**

Better understanding of patient experience is an important driver for service improvements and can act as a lever for system change. In the United Kingdom, the patient experience is now a central issue for the National Health Service Commissioning Board, clinical commissioning groups and the providers they commission from. Traditionally, dementia care in the United Kingdom has focused predominantly on the individual experience of those with late onset dementia, while the voice of those with young onset dementia has been, comparatively, unheard. This study aims to improve the understanding of the personal experience of younger people undergoing investigation for dementia.

**Methods:**

A modified Delphi approach was undertaken with 18 younger people with dementia and 18 supporters of people with young onset dementia. Questions were informed by a scoping review of the literature (O’Malley, M., Carter, J., Stamou, V., Lafontaine, J., & Parkes, J. (2019a). Receiving a diagnosis of young onset dementia: A scoping review of lived experiences. *Ageing & Mental Health*, *0*(0), 1-12). Summary individual statements were refined over two rounds to a final list of 29 key statements.

**Results:**

Twenty-seven of these statements were rated as absolutely essential or very important and included (1) for the general practitioner to identify dementia in younger people, (2) clinicians should be compassionate, empathic and respectful during the assessment and particularly sensitive when providing information about a diagnosis, and (3) remembering that receiving the diagnosis is a lot to absorb for a person with dementia and their supporter. Statistical analyses found no difference in the scoring patterns between younger people with dementia and supporters, suggesting similar shared experiences during the diagnostic process.

**Conclusion:**

Understanding the uniquely personal experience of young people going through the process of diagnosis for dementia is essential to provide person-centred, needs-led, and cost-effective services. Patient’s values and experiences should be used to support and guide clinical decision-making.

## Introduction

The prominence of the ‘patient experience’ as the fourth of five domains in the National Health service (NHS) Outcomes Framework (NHS Digital, 2019) highlights that the patient experience has become a central issue for the NHS Commissioning Board, clinical commissioning groups and the providers they commission from. A better understanding of patient experience can drive service improvements and act as a lever for system change, but at an individual level, it is crucial to provide health care which is person-centred and meets emotional and physical needs. The King’s Fund in 2011 (Robert et al., 2011b) documented that providing the right care the first time around and reducing multiple assessments improves the patient experience in the NHS and avoids unnecessary expenditure. Delivering exceptional patient experience requires the optimising of staff interactions with patients and families and support for staff through ongoing education, training and development.

People with young onset dementia (YOD) face inequity across the dementia pathway compared to those with late onset dementia. This includes taking longer to get an accurate and specific diagnosis, a lack of age appropriate services and a lack of support to meet their unique needs (Rodda & Carter, 2016; Svanberg et al., 2011; Van Vliet et al., 2013). Capturing what matters to younger adults diagnosed with dementia undergoing assessment for dementia is currently lacking (O’Malley et al., 2019a). International research shows that for these young people, aged below 65 years, receiving a confirmed and accurate diagnosis of dementia can be a long and daunting process, taking on average up to 4 years in the Netherlands (Van Vliet et al., 2013; Vernooij-Dassen, 2006) and 4.7 years in Australia (Draper et al., 2016). Compared with late onset dementia (dementia diagnosed over the age of 65 years), the presentations of YOD are likely to be of rare cause disorders, and the common dementias (such as Alzheimer’s disease) frequently present with atypical symptoms (such as visual loss as seen in Alzheimer’s disease variant of posterior cortical atrophy) rather than with memory loss as the first symptom (Harding et al., 2018; Rosness et al., 2016; Vieira et al., 2013).

The increased frequency of symptoms, other than memory loss, upon first presentation tends to result in misdiagnoses, such as psychiatric disorders, depression or other neurological illness (Vieira et al., 2013). Even when presentations include complaints about memory loss, the lack of YOD awareness amongst some healthcare professionals can result in a late detection of red flag symptoms and an under recognition that dementia could be the underlying cause of the symptoms. This period is coupled with feelings of uncertainty for families and a delay in accessing suitable support (Williams et al., 2001). Timely and accurate diagnoses as well as increased awareness of YOD amongst healthcare professionals would help mitigate these issues (Millenaar et al., 2016; Sansoni et al., 2016).

Qualitative studies involving younger people with dementia (YPD) have illuminated how personal and individual the diagnostic journey is (Rabanal et al., 2018; Roach et al., 2016; Wawrziczny et al., 2016). A recent literature review (O’Malley et al., 2019a) has highlighted that delays in diagnosis can be attributed to the initial delays in accessing help by the younger person and the misattribution of symptoms by the clinician. The review also illuminated how reactions to the diagnosis can range from feelings of reassurance (in that their symptoms are now explained) to shock and destabilisation. In addition, the review emphasised how unique the impact of receiving a diagnosis is to each family affected, and how vital the role of the clinician in communicating the diagnosis.

Although a body of research has emphasised qualitative aspects of the experience of diagnosis for young people with dementia (O’Malley et al., 2019a), no research to date has employed a quantitative method aimed at generating and collating the important aspects of the individual experience during the referral, assessment and diagnosis of dementia in a younger adult.

The present study forms part of the evidence for ongoing research conducted by the authors, aimed at improving the quality of diagnosis for YPD (UCL, 2016). The design of the study is a modified Delphi approach in which people living with YOD and their supporters living in England were consulted. In order to further inform this under-researched field, the Delphi process described here was modified to suit the needs of our participants. The findings will provide unique tenets for a code of best practice against which services can be benchmarked.

## Method

### Study design

#### Steering group

The decision to conduct a Delphi study with people living with YOD and their family supporters came from a meeting with the Angela Project’s steering group committee. The Angela Project study design originally included a Delphi study with clinical experts in diagnosis of YOD. Re-evaluation by the research team and steering group committee about the study aims concluded that balance must be provided by additional consultation with experts by experience to understand their personal views about the experience of diagnosis. This led to the current Delphi study format, which has been appropriately adapted to accommodate the unique needs of this specific group.

#### Public and patient involvement group

In line with the CO-researcher INvolvement and Engagement in Dementia Model (Swarbrick et al., 2016), the Patient and Public Involvement (PPI) group for our study was an integral part of the project. The Angela Project’s PPI group was involved from the beginning through to the dissemination phase of the project (Oliver et al., 2020).

#### Literature review

An in-depth literature review (O’Malley et al., 2019a) was conducted to provide focus for the questions and the modified Delphi study design. The review identified eight qualitative research studies which highlighted the key diagnostic concerns for those with YOD as a theme or finding. The review clearly indicated that there was a need for a study specifically focusing on the diagnostic journey.

#### Delphi method

The Delphi method is particularly useful in situations where existing literature is incomplete and inconsistent (Hasson et al., 2000; Keeney et al., 2006). It involves a structured process of collecting information on a specific subject or problem from a panel of experts through a series of questionnaires. The approach allows anonymised individuals to freely express their opinions, reconsider them in the light of collective opinions from the whole group and initiate a narrowing of the range of opinions with each round to gain consensus. As the study focused on an under-studied area, involving a group whose voices are often not heard, we undertook a qualitative first round to capture the experiences and views of our participants (Iqbal & Pipon-Young, 2009; Van Der Steen et al., 2014). Whilst there are shared experiences across individuals and families during the diagnostic journey, receiving a diagnosis of dementia is a unique experience. With this in mind, we modified the Delphi method to include all statements in the final list (including those where consensus was not reached) with their corresponding descriptive statistics to ensure all views were reported and not discarded. In addition, we also offered an e-Delphi option to enable our participants to complete the process online to suit their personal circumstance.

In the present study, the Delphi process to determine what constitutes a good diagnostic experience for YPD involved four steps: (1) formation of the expert panels, (2) survey development informed by a literature search, (3) data collection and analysis and (4) guidelines development.

### Sample selection

The Delphi expert panel consisted of our participants who were younger people living with dementia and family supporters of younger people living with dementia. Previous Delphi studies have had expert panels that have ranged in size from employing five, to more than 60 people, with little evidence to suggest that sample size has any effect on validity or reliability (Powell, 2003). Thirty-six participants (18 people living with dementia and 18 family supporters) took part in Round 1 and 24 participants (11 people living with dementia and 13 family supporters) took part in Round 2, 10 of whom were dyads. Dropout (12 participants in total) was predominantly due to changes in personal circumstances. All participants were recruited from six NHS locations from across England and through national third sector organisations, including the Young Dementia Network (Table 1).

**Table 1.** Participants’ demographic table.

	Demographic		
Person with young onset dementia		Sum	Percentage %
Gender	Female	6	33.33
	Male	12	66.67
Age at diagnosis (mean, SD and range)	61.66 years (SD = 4.02 years). Age range = 39–64 years		
Dementia diagnosis			
	Alzheimer’s disease	7	38.89
	PCA	3	16.67
	FTD	2	11.11
	Mixed dementia – Lewy body, Parkinson’s disease and FTD	1	5.56
	Mixed dementia – Alzheimer’s and FTD	1	5.56
	Vascular dementia	1	5.56
	PPA semantic variant	1	5.56
	Lewy body dementia		5.56
	Short-term memory loss	1	5.56
Previous misdiagnosis			
	Depression	5	27.78
	Epilepsy	3	16.67
	Anxiety	2	11.11
	Stress	2	11.11
	Lifestyle changes	1	5.56
	Thyroid levels	1	5.56
	Bang on the head	1	5.56
	Another dementia diagnosis	1	5.56
	Mild cognitive impairment	1	5.56
Family supporter			
Family supporter gender	Female	14	77.78
	Male	4	22.22
Family supporter type			
	Wife	9	50.00
	Husband	5	27.78
	Partner	1	5.56
	Daughter	1	5.56
	Sister	1	5.56

PCA: posterior cortical atrophy; FTD: frontotemporal dementia; PPA: primary progressive aphasia.

### Survey development

Open-ended questions for Round 1 of the Delphi related to the personal experience of participants about referral, assessment and diagnosis of dementia (see Supplemental Appendix 1 for the questions presented in Round 1) and were co-designed with YPD and family supporters who were members of the PPI panel.

The PPI group were asked to comment and revise the wording of open ending questions for Round 1, and provided feedback on how user-friendly and legible the questionnaires were for both Rounds 1 and 2.

### Analysis framework

The primary aim in the analysis framework was to capture the voices of people with dementia and their supporters. The analysis of Round 1 of the Delphi adopted a structured approach to collate the qualitative responses. Similar responses were therefore grouped and an overarching statement was used to represent the theme. Please see Supplemental Appendix 1 for the questions asked in the first round of the Delphi and Table 2 for the analysis plan for the first round.

**Table 2.** Full list of statements and the supporting raw data quote from the participants with young onset dementia and the family supporters, following Round 1 of the Delphi and in preparation for Round 2.

Diagnostic phase	Statement	Person with dementia quote	Supporter quote
Referral process	For the GP to identify dementia in younger people	*GP reluctant to take seriously as a younger person (115)*	
	Ensure there is enough notice between appointment letters being issued and the appointment	*To ensure there is enough time between appointment letters being issued and the appointment (107)*	
	Making appointments convenient for working adults		*as we were both still working, evening appointments would have been better (229)*
			*It’s important to be supported by your employer who can support you to take time off to accompany your partner to appointments (201)*
			*To be mindful that supporters of people with YOD are still in full-time work, and require time off for appointments (214)*
			*Being mindful that supporters of people with YOD are still in full-time work, and some can only come to appointments on certain days of the week which can prolong delays (226)*
		*The fact that I had the brain scan on a Saturday was very helpful (129)*	
	Being kept in the loop and feeling involved in the assessment	*I felt that the communication was good that everyone I had seen was in the loop (103)*	*I was pleased information was being shared between health professionals – gave me a form of confidence (207)*
		*I felt involved during the assessment and diagnosis (125)*	*Making sure the person and their family are kept in the loop (202)*
			*It’s important to keep the person with dementia and their family in the loop (207)*
			*Fully explaining each of the processes in the diagnosis (205)*
	Healthcare professionals should make contact with family supporters if unable to get through to the person with dementia directly regarding appointments		*It would have been better if my number had been given to the clinic and not my husbands. After all, he was suspected of having a memory problem – would he remember that they had called or what the clinic had said to him (222)*
	The clinicians should listen to the person with dementia and their family as a whole		
		*Being believed and listened to (130). Believing me that something was wrong and not saying it was all down to stress (133)*	
			*In case of dementia, where patients have little or no awareness of their symptoms their carer/partner experiences extreme distress because they cannot get the sufferer to be assessed by the GP. NB It would be so much better if a concerned relative could request a GP to do an assessment in the house. With frontotemporal dementia/behavioural variant the sufferer has no idea that anything is wrong! This creates emotional anguish and distress for the family as their hands are tied!!! (204)*
			*I feel that if we were listened to as a family who know my husband inside out, he might have got his diagnosis earlier and started medication earlier (202)*
		*I would have liked to have been just listened to (102)*	*listen to the family as a whole (202)*
		*if the doctors took into account the families worries and not just the person with dementia as well all know depending on how they are feeling on any given day, the answers, and how they present can be very different (202)*	*if the doctors took into account the families worries and not just the person with dementia as well all know depending on how they are feeling on any given day, the answers, and how they present can be very different (202)*
	Having an identified key person as a single point of contact throughout the whole diagnostic process		*It’s important to have a point of contact between appointments when appointments are several months apart (203)*
			*Consultations speeded up instead of months in between (205)*
		*Having someone to contact when you have a specific question (203)*	*For referrals to be made to specialist units. Better awareness and training in Mental Health Trusts on the issues faced by younger people with dementia (224)*
		*Having someone, who was involved in your diagnosis, who you can call and speak to when necessary (112)*	
	Communication with clinicians should ideally be in person	*Speak with specialists in person rather than on the phone (105)*	
		*Face to face interactions were good (115)*	
		*Very respectful service. Good communication (124)*	
		*Communication by Neurologist was good (125)*	
	Avoid the same questions being asked by the separate clinicians where possible	*Avoid the same questions being asked by the separate clinicians (107)*	
			*It felt like we kept repeating the same things over and over again (201)*
Assessment process	The referral process from GP to first assessment needs to be shorter	*GP making a quick referral to the neurology unit (124)*	*Making a quick referral to the most appropriate specialist (214)*
			*Having timely appointments. A friendly welcome (205)*
		*Not wait 3 months to see the neurologist (125)*	*Referral to a specialist centre straight away this would of save months of upset and distress and unbearable waiting (224)*
			*A much earlier referral ... in view of presentation of symptoms (230)*
		*Being sign-posted to the correct department earlier (130). Not having to go around in circles would have been helpful. It was also very exhausting and confusion having a big black cloud after us (112)*	*Not having to go around in circles – it was also very exhausting and confusing having a black cloud after us (212)*
	Referrals should ideally be made to specialist YOD clinicians and services		*I would of preferred not to of been seen in older persons MH (224)*
		*Making sure referrals to specialist services are made, who can provide helpful information (124)*	*For referrals to be made to specialist units. Better awareness and training in Mental Health Trusts on the issues faced by younger people with dementia (224)*
			*To only make referrals to specialist services (224)*
			*By Direct referral to specialists who know what they are doing and are equipped to advise and support you (224)*
	Clinicians should be compassionate, empathic and respectful during the assessment and particularly sensitive when providing information about a diagnosis		*The first assessment (local neurologist looking at MRI scans) was extremely blunt and distressing. "sorry my dear, this is going to destroy you" (204)*
			*More sensitive handling of devastating news/emotional support (204)*
		*Being aware that people may be anxious about receiving their results (115)*	
			*sympathetic ears are always welcoming (227)*
			*Delivering the diagnosis with compassion and respect (204)*
			*It is so important to that you feel people understand what you are going through (otherwise you feel isolated and fending for yourself) (204)*
			*The diagnosis was handled sympathetically and very re-assuring (227)*
			*Being tactful and careful when discussing how quickly the person’s dementia may deteriorate (201)*
		*Questions were answered using clear language, not jargon (115)*	
		*Questions were answered truthfully (102)*	
	To be seen at home for assessments and post-diagnostic support where appropriate	*Felt relaxed and comfortable with neuropsychology – seen at home (115)*	*A neuropsychologist came out from the XXX memory assessment service. Seen at home, reassuring, pleasant (215)*
		*Being seen in own surroundings, made me feel more relaxed (115)*	*Being seen at home. More relaxed and informal (214)*
		*Being seen at home was helpful (114)*	*Maybe a home visit would have been more appropriate rather than the NHS clinic. Seeing other people with different health problems could have a stigma effect (227)*
		*Since diagnosis they have all been very helpful, especially the admiral nurse that comes to our home to see us (102)*	
	Giving the person with dementia and their family enough opportunities to ask questions		
		*Giving the patient and their family enough opportunities to ask questions (105)*	*The consultant really took their time to explain things and answer any questions (204)*
	Clinicians should be calm, approachable and easy to talk to	*Staff being easy to speak to make you feel more welcome in the memory centre (105)*	*Having a relaxed approach. (215). Support from the nurses at the memory clinic – never feeling “alone" or that it was “my problem" – Feeling like I can call at anytime (205). A good and sympathetic opinion (219)*
			*As a clinician, being easy to talk too, and having a calming approach (201)*
	Clinicians should offer opportunities for the person with dementia and their supporters to speak separately about any issues they wish to discuss		*Taking both the person with dementia’s and the family supporters views separately as these views can be quite different (202)*
		*My partner would have liked to ask the questions without me being there (130)*	
			*Offering to speak to the person with dementia and the carer individually, should they have any questions they would like to ask in private (230)*
	To have a multidisciplinary team involved in diagnosis to provide appropriate support		*Having an OT who specialises in dementia to be allocated to us to help us understand and to give us advice (233)*
			*The opportunity to see other professionals besides neurologists (230)*
		*Having a supportive team of specialists to address questions and fears is important (119)*	
			*Provide better explanations of processes rather than recommending booklets alone. (207) Better information and support through the process (225)*
	More awareness and training on rarer dementia types as well as the issues faced by younger people with dementia in mental health trusts		*For referrals to be made to specialist units. Better awareness and training in Mental Health Trusts on the issues faced by younger people with dementia (224)*
			*More awareness of PCA amongst medical professionals*
	Being understanding during the assessments, especially visual tests for people with PCA	*Being understanding during the visual tests, especially for people with PCA (130)*	*More understanding of PCA and the problems it presents (230)*
	Assessments should be conducted in a quiet and private room		*To have privacy, and a quiet, private room during the assessments (227)*
	Having more information on what the SPECT scanning was all about	*Having more information on what the SPECT scanning was all about (133)*	*A better explanation of why certain tests need to be done (207)*
	Better access to sleep and anger clinics		*Having better access to sleep and anger clinics. Having referrals made to units more local (212)*
	The MRI experience should provide blankets, ear protectors to reduce noise and allow supporters to be in the room if the person wishes	*Reducing noise during the MRI scan (125)*	*Provide ear protectors and a blanket during MRI (225)*
		*Providing an option for supporters to come into the MRI room for support (105)*	
		*Providing blankets during MRI scanning is important (125)*	*Provide ear protectors and a blanket during MRI (225)*
	Results to be given in clinic more quickly	*Perhaps results to be given in clinic more quickly (124)*	
	The time taken to achieve a formal diagnosis needs to be shortened if possible	*A time span of 6 months to diagnosis is acceptable (103)*	
		*Shorter time from start to finish of the diagnosis (125)*	
		*The process could have been improved if it could have been diagnosed quicker and again if they listened to us as a family. (102). Shortening the time to diagnosis (125)*	*Needed to be quicker overall, from GP visit initially to the diagnoses (233)*
	Providing the people with dementia and their families with information about their diagnosis and prognosis if they wish it	*Being told the potential life expectancy was helpful (133)*	*Being told what could happen as we progress down the time-line is helpful (233)*
			*The doctor should fully explain the type of dementia that’s been diagnosed and prognosis (225)*
Diagnosis process	Clinicians should explain medical terms, and what they mean in a simplified manner	*He used the medical terms but fully explained what this meant (103). No but then my wife has always been very good at explaining things to me and others in lay terms (129)*	*All reference to the diagnosis has difficult terminology but this was explained and having the follow up letter I was able to research and look up the reference (203)*
			*The doctor was very honest and explained everything in a language we could understand (202)*
		*To explain that possibility of being on a placebo rather than drug when participating in research projects (112)*	
	Remembering that receiving the diagnosis is a lot to take in for the person with dementia and supporter	*Remembering that it is a lot to take in for the person with dementia (114)*	
			*Going online, I’ve come to understand the diagnosis a lot better (202). Being given some websites to visit with people’s experiences of dementia is helpful (203)*
	Providing the person with dementia and their supporters with a letter which details the diagnosis	*Delivering the diagnosis face-to-face, face and providing a subsequent letter (102)*	*ll sp reference to the diagnosis has difficult terminology but this was explained and having the follow up letter I was able to research and look up the reference (203)*

PCA: posterior cortical atrophy; YOD: young onset dementia; SPECT: single photon emission computed tomography; MRI: magnetic resonance imaging; GP: general practitioner.

## Round 1

The analysis framework for Round 1 consisted of 4 stages.

### Stage 1

The first stage focused on the researchers’ familiarisation with the qualitative responses from our participants and involved. The researchers read through all reports from the participants and where appropriate grouped the exact quotes from that reported similar topics. Quotes were revised and rewritten to develop a summary short title (please see Supplemental Appendixes 2 and 3 for the short titles), and a longer detailed title, for clarity and legibility and a second checker read through the statements and prepared feedback. Only the detailed longer titles are included in the main body of this article. Finally, the second checker and researcher attended a ‘statement workshop’ where statements were grouped and collapsed as appropriate.

Following this Stage 1 process, there were 224 statements in total. One hundred statements were from people with dementia and 124 statements were from supporters.

### Stage 2

Two of the researchers collated similar statements per question across the two groups of YPD and supporters, further reducing the statements to 81 in total. These were next itemised as originating either from both people with dementia and supporters or separately from people with dementia or supporters.

### Stage 3

Similar statements were further reduced by looking at similarities across the whole dataset. Doing this reduced the list of Delphi statements to a final list of 29. See Table 2 for the final list of statements and the supporting quotes from YPD and family supporters.

### Stage 4

Statements were organised according to three headings; referral, assessment and diagnosis of YOD. Consultation with the project PPI members, between February 2017 and December 2019, provided guidance on how best to present the statements to participants in the final round. This consultation included the presentation of the rating scale, font type and size and wording of the statements.

## Round 2

In the final round (Round 2) of the Delphi, participants were asked to rate the importance of the 29 statements using a 7- point Likert scale, with points on the scale representing whether statements were not at all important, low importance, slightly important, neutral, moderately important, very important or absolutely essential. In Round 2, we also wanted to explore whether there were any statistically significant differences in the Likert scale ratings between those with YOD versus family supporters.

### Ethics

The Angela Project was approved by the Health Research Authority in England and by the South Central Berkshire Research Ethics Committee (REC ref.: 17/SC/0296).

## Findings

**Figure 1.**
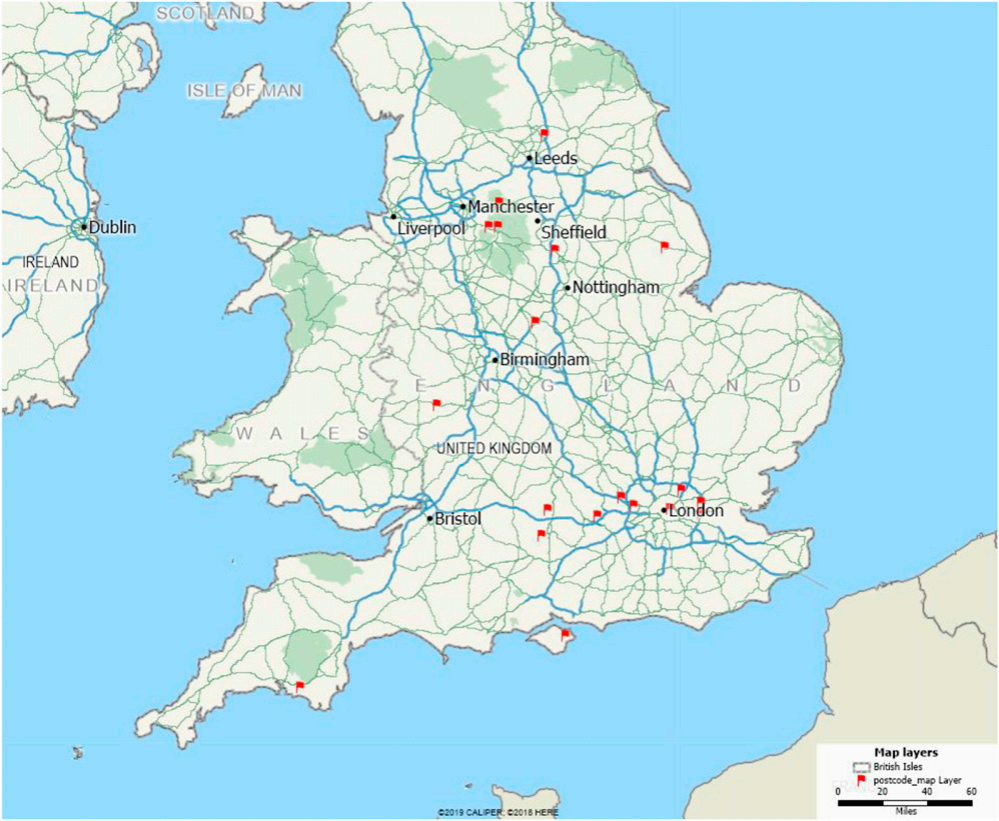
Geographical spread of participants who took part in the Delphi study. This image was produced by the research team using Maptitude 2019 (Calibre Corporation).

Thirty-six participants, 18 people diagnosed with YOD and 18 family supporters were recruited between February 2018 and July 2018. See Figure 1 below which shows the geographical locations of the participants.

### Location

Two rounds of a modified Delphi process resulted in 29 key statements related to referral, assessment and diagnosis of which 27 were rated by participants as absolutely essential or very important. Please see Table 2 for the full list of 29 of statements that were organised following the analysis framework of Round 1 of the modified Delphi and the supporting raw data quote from the participants with YOD and the family supporters.

#### Statistical analyses

In addition to the rich qualitative data to support the formation of each statement, we wanted to explore whether there were any significant differences in the ratings given by those with YOD versus family supporters following Round 2. The distributions of the ratings for all 29 statements were non-normal; therefore, a non-parametric test (Mann–Whitney test) was used for the analysis. Statistical significance was tested at the 5% level throughout.

Table 3 consists of the full list of the statements, inter-quartile range, median score and results of the Mann–Whitney test. The two statements highlighted denoted ** have averages which are moderately important.

**Table 3.** Full list of the statements, inter-quartile range, median score and results of the Mann–Whitney test that compared ratings between people with dementia and family supporters.

Diagnostic phase	Statement	Respondent	Rating			Mann–Whitney test	
			Lower quartile	Median	Upper quartile	U	*p*
Referral process	For the GP to identify dementia in younger people	People with dementia	6.00	7.00	7.00	59.00	.39
		Supporter	6.00	7.00	7.00		
	Ensure there is enough notice between appointment letters being issued and the appointment	People with dementia	5.00	5.00	6.50	65.50	.71
		Supporter	5.00	6.00	6.00		
	Making appointments convenient for working adults	People with dementia	5.00	6.00	6.00	53.50	.26
		Supporter	6.00	6.00	7.00		
	Being kept in the loop and feeling involved in the assessment	People with dementia	6.00	7.00	7.00	62.50	.55
		Supporter	6.00	6.00	7.00		
	Healthcare professionals should make contact with family supporters if unable to get through to the person with dementia directly regarding appointments	People with dementia	6.00	7.00	7.00	67.50	.78
		Supporter	6.00	7.00	7.00		
	The clinicians should listen to the person with dementia and their family as a whole	People with dementia	6.00	7.00	7.00	66.00	.72
		Supporter	6.00	7.00	7.00		
	Having an identified key person as a single point of contact throughout the whole diagnostic process	People with dementia	6.00	6.00	7.00	66.00	.71
		Supporter	6.00	6.00	7.00		
	Communication with clinicians should ideally be in person	People with dementia	6.00	6.00	7.00	71.00	.97
		Supporter	6.00	6.00	7.00		
	Avoid the same questions being asked by the separate clinicians where possible	People with dementia	5.50	6.00	7.00	60.50	.50
		Supporter	5.00	6.00	7.00		
Assessment process	The referral process from GP to first assessment needs to be shorter	People with dementia	6.00	6.00	7.00	63.00	.56
		Supporter	6.00	7.00	7.00		
	Referrals should ideally be made to specialist YOD clinicians and services	People with dementia	6.00	7.00	7.00	59.00	.39
		Supporter	6.00	7.00	7.00		
	Clinicians should be compassionate, empathic and respectful during the assessment and particularly sensitive when providing information about a diagnosis	People with dementia	6.00	6.00	7.00	52.00	.18
		Supporter	7.00	7.00	7.00		
	To be seen at home for assessments and post-diagnostic support where appropriate	People with dementia	5.00	5.00	6.00	71.00	.98
		Supporter	5.00	5.00	6.00		
	Giving the person with dementia and their family enough opportunities to ask questions	People with dementia	6.00	7.00	7.00	61.50	.52
		Supporter	6.00	6.00	7.00		
	Clinicians should be calm, approachable and easy to talk to	People with dementia	6.00	7.00	7.00	66.00	.72
		Supporter	6.00	7.00	7.00		
	Clinicians should offer opportunities for the person with dementia and their supporters to speak separately about any issues they wish to discuss	People with dementia	6.00	6.00	7.00	49.50	.15
		Supporter	6.00	7.00	7.00		
	To have a multidisciplinary team involved in diagnosis to provide appropriate support	People with dementia	6.00	6.00	6.50	68.00	.81
		Supporter	6.00	6.00	7.00		
	More awareness and training on rarer dementia types as well as the issues faced by younger people with dementia in mental health trusts	People with dementia	6.00	7.00	7.00	62.50	.51
		Supporter	7.00	7.00	7.00		
	Being understanding during the assessments, especially visual tests for people with PCA	People with dementia	6.00	6.00	7.00	57.50	.36
		Supporter	6.00	7.00	7.00		
	Assessments should be conducted in a quiet and private room	People with dementia	6.00	6.00	7.00	55.50	.29
		Supporter	6.00	7.00	7.00		
	Having more information on what the SPECT scanning was all about	People with dementia	5.00	7.00	7.00	61.50	.49
		Supporter	6.00	7.00	7.00		
	Better access to sleep and anger clinics	People with dementia	5.00	6.00	6.00	68.50	.86
		Supporter	4.00	6.00	6.00		
	The MRI experience should provide blankets, ear protectors to reduce noise and allow supporters to be in the room if the person wishes	People with dementia	5.50	6.00	7.00	68.00	.83
		Supporter	5.00	6.00	7.00		
	Results to be given in clinic more quickly	People with dementia	6.00	6.00	7.00	67.50	.80
		Supporter	6.00	6.00	7.00		
	The time taken to achieve a formal diagnosis needs to be shortened if possible	People with dementia	6.00	7.00	7.00	64.00	.62
		Supporter	6.00	7.00	7.00		
	Providing the people with dementia and their families with information about their diagnosis and prognosis if they wish it	People with dementia	6.00	7.00	7.00	63.50	.59
		Supporter	6.00	7.00	7.00		
Diagnosis process	Clinicians should explain medical terms, and what they mean in a simplified manner	People with dementia	6.00	7.00	7.00	66.50	.74
		Supporter	6.00	7.00	7.00		
	Remembering that receiving the diagnosis is a lot to take in for the person with dementia and supporter	People with dementia	6.00	7.00	7.00	58.00	.34
		Supporter	7.00	7.00	7.00		
	Providing the person with dementia and their supporters with a letter which details the diagnosis	People with dementia	6.00	7.00	7.00	58.00	.34
		Supporter	7.00	7.00	7.00		

PCA: posterior cortical atrophy; YOD: young onset dementia; SPECT: single photon emission computed tomography; MRI: magnetic resonance imaging; GP: general practitioner.

The statement ‘Ensure there is enough notice between appointment letters being issued and the appointment’ only reached a moderate importance consensus level for people with dementia and ‘To be seen at home for assessments and post-diagnostic support where appropriate’ only reached moderate importance consensus level for both people with dementia and family supporters.

#### Statistics

There were no statistically significant differences between statements expressed by the people with dementia and their supporters. There was a ceiling effect which effectively decreased the sensitivity of the scale since most of the median ratings were 7 (Absolutely essential), with the lowest median rating being 5 (Moderately important). However, this does show a high degree of agreement that the statements extracted were considered important to all participants. Paired analysis of the ratings of people with dementia and their supporter also did not show any statistically significant differences in ratings for any of the statements.

### Agreement between those diagnosed with dementia and supporters

Following the ratings made for each statement in Round 2, scores were available for 10 dyads who participated in this round. Paired tests (Wilcoxon tests) on data from people with dementia/supporter dyads also showed no statistically significant differences between the scores of YPD and their supporters on statements, with the exception of the statement ‘Making appointments convenient for working adults’ where there was a statistically significant difference between the responses of people with dementia compared to their supporters, with the supporters generally reporting this as having higher importance (related-samples Wilcoxon test, test statistic = 15, *n* = 10, *p* = .038).

### Percentage agreement

When comparing agreement in scoring for all 10 dyads, we found a difference in scoring patterns on aspects of the referral, assessment and diagnosis. Please see Table 4 below for the percentage agreement per statement.

**Table 4.** Level of agreement (percentage) on statements between the 10 dyads that completed all rounds of the Delphi.

Diagnostic phase	Statement	% Agreement
Referral process	For the GP to identify dementia in younger people	90
	Ensure there is enough notice between appointment letters being issued and the appointment	40
	Making appointments convenient for working adults	50
	Being kept in the loop and feeling involved in the assessment	70
	Healthcare professionals should make contact with family supporters if unable to get through to the person with dementia directly regarding appointments	50
	The clinicians should listen to the person with dementia and their family as a whole	60
	Having an identified key person as a single point of contact throughout the whole diagnostic process	70
	Communication with clinicians should ideally be in person	80
	Avoid the same questions being asked by the separate clinicians where possible	30
Assessment process	The referral process from GP to first assessment needs to be shorter	40
	Referrals should ideally be made to specialist YOD clinicians and services	60
	Clinicians should be compassionate, empathic and respectful during the assessment and particularly sensitive when providing information about a diagnosis	60
	To be seen at home for assessments and post-diagnostic support where appropriate	60
	Giving the person with dementia and their family enough opportunities to ask questions	70
	Clinicians should be calm, approachable and easy to talk to	60
	Clinicians should offer opportunities for the person with dementia and their supporters to speak separately about any issues they wish to discuss	50
	To have a multidisciplinary team involved in diagnosis to provide appropriate support	40
	More awareness and training on rarer dementia types as well as the issues faced by younger people with dementia in mental health trusts	80
	Being understanding during the assessments, especially visual tests for people with PCA	80
	Assessments should be conducted in a quiet and private room	70
	Having more information on what the SPECT scanning was all about	60
	Better access to sleep and anger clinics	30
	The MRI experience should provide blankets, ear protectors to reduce noise and allow supporters to be in the room if the person wishes	50
	Results to be given in clinic more quickly	70
	The time taken to achieve a formal diagnosis needs to be shortened if possible	90
	Providing the people with dementia and their families with information about their diagnosis and prognosis if they wish it	70
Diagnosis process	Clinicians should explain medical terms and what they mean in a simplified manner	70
	Remembering that receiving the diagnosis is a lot to take in for the person with dementia and supporter	70
	Providing the person with dementia and their supporters with a letter which details the diagnosis	40

PCA: posterior cortical atrophy; YOD: young onset dementia; SPECT: single photon emission computed tomography; MRI: magnetic resonance imaging; GP: general practitioner.

It is important to note that the percentage agreement between dyad findings do not take into account the agreement that would be expected purely by chance. High levels of agreement do not mean high levels of importance of that statement (just that most pairs of people with dementia gave the same score for that statement as their supporter). Note that there were no statistical differences between the paired scores for all but one of the statements, so low percentages do not suggest that people with dementia scored differently overall to their supporters (the differences were in both directions; sometimes people with dementia scored higher than supporters, sometimes the other way round).

### Discussion

In this study, young people with dementia and their supporters have highlighted key components of the referral, assessment and diagnosis that they deem to be absolutely essential or very important for informing best practice based on their own personal experience.

People with YOD expressed concern about inequity in waiting times in receiving a diagnosis and access to necessary investigations, as highlighted in the present study’s final list of 29 statements. Research has shown people with YOD can wait 4 years (Van Vliet et al., 2013) for diagnosis, and that in England, only 45.9% of those predicted to have a diagnosis of YOD have a recorded diagnosis compared to those over 65 where the recorded diagnosis rate is 68% (Public Health England, 2020)*.*

In general practice, delays may be due to general practitioners (GPs) not considering the possibility of dementia in younger people and because the rarer types of dementia that are more common in younger people are harder to recognise and have symptoms that overlap with those of common psychiatric disorders such as depression. This explanation is consistent with the reports of misdiagnosis by the participants in the current study, whereby 15 of the 18 individuals reported a diagnosis of another condition before receiving a confirmed diagnosis of dementia. Once someone is referred to a specialist setting, there can be further delays due to a lack of specialist clinicians and limited access to the often, complex investigations required to diagnose YOD. This means a longer period of having to cope with unexplained symptoms and no support, for both the person with dementia and their family.

Healthcare research has established that involving individuals in shared decision-making by encouraging active participation and enhanced communication, can provide individuals with more control over their care, improves the ability to make informed choices and allows them to participate knowledgeably in treatment decisions (De Wilde et al., 2017; Elwyn et al., 2010). Shared decision-making in dementia care is a relatively new concept (Mariani, 2019) and has more often been implemented in terms of care planning and end-of-life care (Gjerberg et al., 2015), though more recently research is exploring shared decision-making during the diagnostic process (De Wilde et al., 2017). As captured in our statements, patient–clinician conversations during the workup require sensitivity, and care should be taken when delivering updates on ongoing assessments and when delivering diagnoses.

Evidence suggests that improving the patient experience is linked to improvement in performance and systems within clinical practice (Schlesinger et al., 2015) but equally as important it increases individual autonomy and empowerment to maintain independence (Stamou et al., 2020). The results presented here support this view by clearly demonstrating that while both the efficiency and practicalities of the diagnostic process were important, participants equally valued feeling listened to, informed and supported.

Of note, rapid referral to specialists, early identification of presenting symptoms by GPs, convenient appointment times especially for working adults are in-line with known ‘pinch points’ in current care pathways for YPD which result in delays in referral (O’Malley et al., 2019a, 2019b; Van Vliet et al., 2011). Clinicians taking time to gather the views of important informants and listening to the whole family, overlaps with good practice guidance for clinicians in assessment and history taking, particularly where the person with dementia may lack insight into their difficulties or the presentation is non-amnestic and harder to recognise (Harding et al., 2018; O’Malley et al., 2019b). YPD endorsed the value of having an identified key person as a single point of contact throughout the whole diagnostic process. Although, this approach to case management is enshrined in the National Institute for Clinical Excellence dementia guideline (National Institute for Health and Clinical Excellence, 2018), the necessity for specialist skills in the case management role specifically relevant to YPD are usually not acknowledged. For example having skills and knowledge to facilitate access to information about YOD and rare forms of dementia, to communicate the diagnosis to young children and to facilitate access to specialist advice and support about young onset specific needs, for example employment, mortgage and financial obligations and future financial planning, guidance on this role is available (Hussey & Hayo, 2019).

The communication skills of the clinician and the feeling of being listened to and heard by those with expertise in diagnosis formed the focus of most statements in relation to the assessment stage of the process. Sensitivity about the impact of the information because of the ‘lack of narrative’ for dementia at a young age and making time for questions with follow-up summary information were particularly valued in terms of the way diagnosis was relayed. Getting a diagnosis in working age can significantly disrupt the normal life events, particularly when the person faces increasing disability, dependency and mortality (Clemerson et al., 2013; Pipon-Young et al., 2012). At the point of diagnosis, there would be an opportunity for the clinician to have a conversation with the person about the impact of the diagnosis on the changes they may be faced with, and how they might adjust (Roach et al., 2008). However, these conversations would need to be considered in a person-centred and individual way as they may not be appropriate for some individuals.

The clinician’s use of language, avoiding the use of medical jargon and adopting a calm manner in a private environment were all also valued. This mirrors findings in a recently published scoping review that highlighted how the impact of a diagnosis on the patient and their supporter was heavily influenced by the language used by the clinicians (O’Malley et al., 2019a, 2019b).

Several generic frameworks have attempted to capture what matters most to patients (Robert et al., 2011a) in terms of improving individual experience, and the statements identified here show significant overlap with their core tenets, often identified as relational and functional aspects. Most research in the field of patient experience has focused upon the relational aspects of care (feeling informed, listened to) but interestingly in our study, the majority of statements preferentially related to functional aspects of care (i.e. the process). This may reflect previous research which demonstrates that those with YOD often see up to five different consultants before diagnosis and care pathways can be chaotic (Carter et al., 2018). Our own research which identifies the core features of YOD services which are perceived positively (Stamou et al., 2020) demonstrates that positive post-diagnostic services may collectively create an enabling-protective circle that supports YPD to re-establish and maintain a positive identity in the face of YOD.

It could be argued that many of the individual statements reported by YPD and family member/supporters simply represent good practice in all-age dementia assessment. However, statements related to knowledge base of rare dementias, GP recognition of early symptoms, shortening the time to diagnosis and explanation of specialist investigations, arguably reflect the reality of current shortfalls in services for those with YOD (Murrells et al., 2013). Additionally, the value of the statements here is that they provide insight into the multidimensional aspects of individual experience ranging from ‘relational’ aspects of care, such as feeling informed, listened to, communication styles, to ‘functional’ aspects of care, such as the practicalities of the process and how this can guide shared decision-making, deliver a more person-centred experience and increase individual autonomy.

Interestingly, there were no significant differences in the opinions expressed by YPD or family/supporters, although it is recognised that this may often not be the case. This might be explained in the current study by the low number of participants with dementia subtypes more commonly associated with reduced insight such as frontotemporal dementias.

The statements derived from this Delphi study offer the potential to identify shortfalls in current services and improve the quality of services to better meet the needs of YPD and families.

### Strengths and limitations

Although we recruited a broad geographical spread of participants, only individuals living in England took part in the study. Diagnostic experiences from the rest of the United Kingdom were therefore not captured and were beyond the scope of the current study. Future research should aim to include those living in Wales, Scotland and Northern Ireland to explore whether the statements and key reports are consistent with the experiences of those living in the rest of the United Kingdom and whether additional statements should be considered for other regions.

A modified Delphi methodology was adopted to refine the statements viewed as being crucial during the diagnostic period. Consensus was not the prime aim of this article rather it was to capture absolutely essential and very important aspects of the process of diagnosis for young people with dementia. We have presented the full list of statements to ensure that all views are captured, and statements were not excluded because they represented a minority view. The limited number of participants means that the study may have missed important lived experiences of younger people undergoing assessment for dementia and may not be truly representative. It was also a small sample for statistical analysis and may have not had sufficient power to identify small to moderate differences. However, the population of individuals who participated came from across the whole of England (see Figure 1 for the geographical spread) and were recruited through both NHS services for YPD, as well as third sector organisations and therefore could be considered representative.

How people with dementia experience their condition depends on their own complex biographies and relationships as well as the behaviour of those they encounter during the diagnostic process. Everyone’s experience of receiving a diagnosis of dementia is unique, so practitioners and clinicians should use our findings as guidance but continue to listen to the views of their own patients in their specific setting and be alert to expressed differences.

### Implications and implementation

The qualitatively rich reports made by our participants highlighted key aspects of the referral, assessment and diagnosis of dementia that should be considered by healthcare organisations as important to the individual experience and hence delivery of good care. Good experience is generally considered a multidimensional concept dependent on functional (process), transactional (‘being care for’) and relational (‘being care about’) aspects of care. Several approaches to measurement of these aspects of care are available and future work is necessary to assess how these can inform a strategic approach to improve the experience for young people with dementia and their families/supporters.

## Conclusion

In this article, we have presented the findings from a unique and innovative modified Delphi deliberately designed to capture the perspectives of YPD and their carers as ‘experts’ of their experiences. The study provides insight into the complex interpersonal aspects of care that matter to YPD, alongside transactional and functional aspects that are necessary to improve individual experience.

## Supplemental Material

Supplemental_material – Supplemental Material for Receiving a diagnosis of young onset dementia: Evidence-based statements to inform best practice Supplemental Material, Supplemental_material for Receiving a diagnosis of young onset dementia: Evidence-based statements to inform best practice by Mary O’Malley, Jacqueline Parkes, Jackie Campbell, Vasileios Stamou, Jenny LaFontaine, Jan R Oyebode and Janet Carter in Dementia

## References

[bibr1-1471301220969269] Carter J. E. Oyebode J. R. Koopmans R. T. C. M. (2018). Young-onset dementia and the need for specialist care: A national and international perspective. Aging & Mental Health, 22(4), 468-473. DOI: 10.1080/13607863.2016.1257563. 28290708

[bibr2-1471301220969269] Clemerson G. Walsh S. Isaac C. (2013). Towards living well with young onset dementia: An exploration of coping from the perspective of those diagnosed. Dementia, 13(4), 451-466. DOI: 10.1177/1471301212474149. 24339066

[bibr3-1471301220969269] De Wilde A. van Maurik I. S. Kunneman M. Bouwman F. Zwan M. Willemse E. A. J. Biessels G. J. Minkman M. Pel R. Schoonenboom N. S. M. Smets E. M. A. Wattjes M. P. Barkhof F. Stephens A. van Lier E. J. Batrla‐Utermann R. Scheltens P. Teunissen C. E. van Berckel B. N. M. van der Flier W. M. (2017). Alzheimer’s biomarkers in daily practice (ABIDE) project: Rationale and design. Alzheimer’s & Dementia: Diagnosis, Assessment & Disease Monitoring, 6, 143-151. DOI: 10.1016/j.dadm.2017.01.003. PMC5318541 28239639

[bibr4-1471301220969269] Draper B. Cations M. White F. Trollor J. Loy C. Brodaty H. Sachdev P. Gonski P. Demirkol A. Cumming R. G. Withall A. (2016). Time to diagnosis in young-onset dementia and its determinants: The INSPIRED study. International Journal of Geriatric Psychiatry, 31(11), 1217-1224. DOI: 10.1002/gps.4430. 26807846

[bibr5-1471301220969269] Elwyn G. Laitner S. Coulter A. Walker E. Watson P. Thomson R. (2010). Implementing shared decision making in the NHS. Bmj: British Medical Journal, 341, c5146. DOI: 10.1136/bmj.c5146. 20947577

[bibr6-1471301220969269] Gjerberg E. Lillemoen L. Forde R. Pedersen R. (2015). End-of-life care communications and shared decision-making in Norwegian nursing homes - Experiences and perspectives of patients and relatives. BMC Geriatrics, 15(1), 1-13. DOI: 10.1186/s12877-015-0096-y. 26286070 PMC4544816

[bibr7-1471301220969269] Harding E. Sullivan M. P. Woodbridge R. Yong K. X. X. McIntyre A. Gilhooly M. L. Gilhooly K. J. Crutch S. J. (2018). “Because my brain isn’t as active as it should be, my eyes don’t always see”: A qualitative exploration of the stress process for those living with posterior cortical atrophy. BMJ Open, 8(2), 1-12. DOI: 10.1136/bmjopen-2017-018663. PMC5829605 29439072

[bibr8-1471301220969269] Hasson F. Keeney S. McKenna H. (2000). Research guidelines for the Delphi survey technique. Journal of Advanced Nursing, 32(4), 1008-1015. 11095242

[bibr9-1471301220969269] Hussey J. Hayo H. (2019). Young dementia: The specialist keyworker role. Journal of Dementia Care, 27(3), 25-27.

[bibr10-1471301220969269] Iqbal S. Pipon-Young L. (2009). The Delphi method. The Psychologist, 22(7), 598-600.

[bibr11-1471301220969269] Keeney S. Hasson F. McKenna H. (2006). Consulting the oracle: Ten lessons from using the Delphi technique in nursing research. Journal of Advanced Nursing, 53(2), 205-212. 16422719 10.1111/j.1365-2648.2006.03716.x

[bibr12-1471301220969269] Mariani E. (2019). LET ME PARTICIPATE Using shared Care, to involve persons with dementia in care planning in long-term. the Netherlands: ProefschriftMaken.

[bibr13-1471301220969269] Millenaar J. K. Bakker C. Koopmans R. T. C. M. Verhey F. R. J. Kurz A. de Vugt M. E. (2016). The care needs and experiences with the use of services of people with young-onset dementia and their caregivers: A systematic review. International Journal of Geriatric Psychiatry, 31(12), 1261-1276. DOI: 10.1002/gps.4502. 27271788

[bibr14-1471301220969269] Murrells T. Robert G. Adams M. Morrow E. Maben J. (2013). Measuring relational aspects of hospital Care in England with the “patient evaluation of emotional care during hospitalisation” (PEECH) survey questionnaire. BMJ Open, 3(1), 1-8. DOI: 10.1136/bmjopen-2012-002211. PMC3563120 23370012

[bibr15-1471301220969269] National Institute for Health and Clinical Excellence (2018). Dementia: Assessment, management and support for people living with dementia and their carers. https://www.nice.org.uk/guidance/ng97/resources/dementia-assessment-management-and-support-for-people-living-with-dementia-and-their-carers-pdf-1837760199109. 30011160

[bibr16-1471301220969269] NHS Digital (2019). About the NHS Outcomes framework (NHS OF). https://digital.nhs.uk/data-and-information/publications/ci-hub/nhs-outcomes-framework.

[bibr17-1471301220969269] Oliver K. O’Malley M. Parkes J. Stamou V. La Fontaine J. Oyebode J. Carter J. (2020). Living with young onset dementia and actively shaping dementia research - The Angela project. Dementia, 19(1), 41-48. DOI: 10.1177/1471301219876414. 31875707

[bibr18-1471301220969269] O’Malley M. Carter J. Stamou V. Lafontaine J. Parkes J. (2019a). Receiving a diagnosis of young onset dementia: A scoping review of lived experiences. Aging & Mental Health, 0(0), 1-12. DOI: 10.1080/13607863.2019.1673699. 31647324

[bibr19-1471301220969269] O’Malley M. Parkes J. Stamou V. LaFontaine J. Oyebode J. Carter J. (2019b). Young-onset dementia: Scoping review of key pointers to diagnostic accuracy. BJPsych Open, 5(3), 1-9. DOI: 10.1192/bjo.2019.36. PMC6582217 31530311

[bibr20-1471301220969269] Pipon-Young F. E. Lee K. M. Jones F. Guss R. (2012). I’m not all gone, I can still speak: The experiences of younger people with dementia. An action research study. Dementia, 11(5), 597-616. DOI: 10.1177/1471301211421087.

[bibr21-1471301220969269] Powell C. (2003). The Delphi technique: Myths and realities. Journal of Advanced Nursing, 41(4), 376-382. http://www.embase.com/search/results?subaction=viewrecord&from=export&id=L36478234. 12581103 10.1046/j.1365-2648.2003.02537.x

[bibr22-1471301220969269] Public Health England (2020). Dementia profile. https://fingertips.phe.org.uk/profile-group/mental-health/profile/dementia.

[bibr23-1471301220969269] Rabanal L. I. Chatwin J. Walker A. O’Sullivan M. Williamson T. (2018). Understanding the needs and experiences of people with young onset dementia: A qualitative study. BMJ Open, 8(10), 1-9. DOI: 10.1136/bmjopen-2017-021166. PMC6196838 30344167

[bibr24-1471301220969269] Roach P. Drummond N. Keady J. (2016). ‘Nobody would say that it is Alzheimer’s or dementia at this age’: Family adjustment following a diagnosis of early-onset dementia. Journal of Aging Studies, 36, 26-32. DOI: 10.1016/j.jaging.2015.12.001. 26880602

[bibr25-1471301220969269] Roach P. Keady J. Bee P. Hope K. (2008). Subjective experiences of younger people with dementia and their families: Implications for UK research, policy and practice. Reviews in Clinical Gerontology, 18(2), 165-174.

[bibr26-1471301220969269] Robert G. Cornwell J. Brearley S. (2011a). What matters to patients? Developing the evidence base for measuring and improving patient experience. http://www.wales.nhs.uk/sites3/documents/420/Final Project Report pdf doc january 2012 (2).pdf.

[bibr27-1471301220969269] Robert G. Cornwell J. Brearley S. Foot C. Goodrich J. Joule N. Levenson R. Maben J. Murrells T. Tsianankas V. Waite D. (2011b). What matters to patients? - Developing the evidence base for measuring and improving patient experience. Project report for the department of health and NHS institute for innovation and improvement. The King’s Fund, 1-200.

[bibr28-1471301220969269] Rodda J. Carter J. E. (2016). A survey of UK services for younger people living with dementia. International Journal of Geriatric Psychiatry, 31(8), 951-959. DOI: 10.1002/gps.4402. 26642828

[bibr29-1471301220969269] Rosness T. A. Engedal K. Chemali Z. (2016). Frontotemporal dementia. Journal of Geriatric Psychiatry and Neurology, 29(5), 271-280. DOI: 10.1177/0891988716654986. 27502302

[bibr30-1471301220969269] Sansoni J. Duncan C. Grootemaat P. Capell J. Samsa P. Westera A. (2016). Younger onset dementia. American Journal of Alzheimer’s Disease & Other Dementias, 31(8), 693-705. DOI: 10.1177/1533317515619481. PMC10852741 26888862

[bibr31-1471301220969269] Schlesinger M. Grob R. Shaller D. (2015). Using patient‐reported information to improve clinical practice. Health Services Research, 50, 2116-2154. 26573890 10.1111/1475-6773.12420 PMC5115180

[bibr32-1471301220969269] Stamou V. Fontaine J. L. O’Malley M. Jones B. Gage H. Parkes J. Carter J. Oyebode J. (2020). The nature of positive post-diagnostic support as experienced by people with young onset dementia. Aging & Mental Health, 0(0), 1-9. DOI: 10.1080/13607863.2020.1727854. 32067481

[bibr33-1471301220969269] Svanberg E. Spector A. Stott J. (2011). The impact of young onset dementia on the family: A literature review. International Psychogeriatrics, 23(3), 356-371. DOI: 10.1017/S1041610210001353. 20735892

[bibr34-1471301220969269] Swarbrick C. M. Doors O., Scottish Dementia Working Group; Educate, Davis K. Keady J. (2016). Visioning change: Co-producing a model of involvement and engagement in research (innovative practice). Dementia, 18, 3165. DOI: 10.1177/1471301216674559. 27753612

[bibr35-1471301220969269] UCL (2016). The Angela project. https://www.ucl.ac.uk/psychiatry/angela-project.

[bibr36-1471301220969269] Van Der Steen J. T. Radbruch L. Hertogh C. M. De Boer M. E. Hughes J. C. Larkin P. Francke A. L. Jünger S. Gove D. Firth P. Koopmans R. T. Volicer L. (2014). White paper defining optimal palliative care in older people with dementia: A Delphi study and recommendations from the European Association for palliative care. Palliative Medicine, 28(3), 197-209. DOI: 10.1177/0269216313493685. 23828874

[bibr37-1471301220969269] Van Vliet D. De Vugt M. E. Bakker C. Koopmans R. T. C. M. Pijnenburg Y. A. L. Vernooij-Dassen M. J. F. J. Verhey F. R. J. (2011). Caregivers’ perspectives on the pre-diagnostic period in early onset dementia: A long and winding road. International Psychogeriatrics, 23(9), 1393-1404. DOI: 10.1017/S1041610211001013. 21729410

[bibr38-1471301220969269] Van Vliet D. de Vugt M. E. Bakker C. Pijnenburg Y. A. L. Vernooij-Dassen M. J. F. J. Koopmans R. T. C. M. Verhey F. R. J. (2013). Time to diagnosis in young-onset dementia as compared with late-onset dementia. Psychological Medicine, 43(2), 423-432. DOI: 10.1017/S0033291712001122. 22640548

[bibr39-1471301220969269] Vernooij-Dassen M. (2006) Receiving a diagnosis of dementia. Dementia, 5(3), 397-410. DOI: 10.1177/1471301206067114.

[bibr40-1471301220969269] Vieira R. T. Caixeta L. Machado S. Cardoso S. Adriana Nardi A. E. Arias-Carrión O. Giovanni Carta M. (2013). Epidemiology of early-onset dementia: a review of the literature. Clinical Practice & Epidemiology in Mental Health, 9(1), 88-95. DOI: 10.2174/1745017901309010088. 23878613 PMC3715758

[bibr41-1471301220969269] Wawrziczny E. Pasquier F. Ducharme F. Kergoat M.-J. Antoine P. (2016). From ‘needing to know’ to ‘needing not to know more’: An interpretative phenomenological analysis of couples’ experiences with early-onset Alzheimer's disease. Scandinavian Journal of Caring Sciences, 30(4), 695-703. DOI: 10.1111/scs.12290. 26453315

[bibr42-1471301220969269] Williams T. Dearden A. M. Cameron I. H. (2001). From pillar to post - a study of younger people with dementia. Psychiatric Bulletin, 25(10), 384-387. DOI: 10.1192/pb.25.10.384.

