# Supplementary material for: Receiving a diagnosis of young onset dementia: Evidence-based statements to inform best practice
Source: Dementia (London). 2020 Oct 30;20(5):1745–71. doi: 10.1177/1471301220969269 (PMC8216318; doi:10.1177/1471301220969269)
Supplement: Supplemental_material – Supplemental Material for Receiving a diagnosis of young onset dementia: Evidence-based statements to inform best practice [file Supplemental_material.pdf]

Appendix 1: Questions Round 1 of the Delphi

The Angela Project: Delphi-EXPERIENCE Questionnaire

Thank you very much for agreeing to act as a “Delphi expert” panel member for our study which aims to improve the diagnostic process for people living with young onset dementia. The study will consist of a minimum of three rounds. This is round one.

In this questionnaire we hope to learn more about your experiences of when **you** received a diagnosis of dementia. Our goal is to understand what worked well in the service(s) you received resulting in your diagnosis and what could be improved.

We would welcome your views as a younger person living with dementia, and if you prefer, we encourage you to involve your supporter/family carer to assist you with answering some of the questions.

What will I have to do?

There will be a series of questions about the referral, assessment and diagnostic processes of your diagnosis. Please answer and reflect on your own experiences. You can write as little or as much as you wish.

Please respond to all questions as this means that we can compare results in a consistent way. This should take approximately 30-45 minutes to complete depending on how detailed your responses are.

Thank you for your valuable assistance in completing this questionnaire. If you would like any support in completing the questionnaire, please contact the researcher before starting the questionnaire and she will provide you with the relevant support.

Please insert the participant code number the researcher gave you:
…………

Initial questions:

1. What is your current age?

   ……………………………

2. What was your age when you were diagnosed with dementia?

   ……………………………

3. Please could you state the official diagnosis you were given:
   ………
   ……………………………………………………………………………………………………

4. Before you were given a diagnosis of dementia, were you given other diagnoses as a possible reason for your symptoms?

   (Please circle your response) Yes No

   a. If yes, please state these below:
   ………
   ………
   ………
   ……………………………………………………………………………………………

5. In which town or county of the UK do you live?
   ………
   ………
   ………
   ……………………………………………………………………………………………

Section 1. The Referral Process

6. Please tell us about your experience of the referral from your GP:

……
……
……
……
……
……
……
……
……………………………………………………………………………………………

a. Which aspects were handled well?

……
……
……
……
……
……
……
………………………………………………………………………………………………

b. How could your experience could have been improved?

……
……
……
……
……
……
……
………………………………………………………………………………………………

7. How did the health professionals communicate between each other during your assessment/diagnosis?

....
....
....
....

a. Did this have any effect on you?

....
....
....
....

8. How might the various appointments with dementia specialists have been better managed to suit you? (i.e. location, time of appointment, form of contact).

....
....
....
....
....
....
....
....

Section 2. The Dementia Assessment Process

9. Please tell us about the assessment you received from the dementia specialist(s)?
…… …… …… …… …… …… …… …………………………………………………………………………………

a. Can you comment on what was helpful?
…… …… …… …… …… …… ……………………………………………………………………………………

10. Based on your experience, is there anything else you would have liked to happen during this assessment process?
…… …… ……

....
....
....
....
....

11. How helpful were the staff you met during the assessment process?

....
....
....
....
....
....
....
....

12. How long did it take from being referred onto a dementia specialist by the GP to receiving your diagnosis of dementia? Please circle the corresponding time-frame:

- **0-6 months**
- **6-12 months**
- **12 months – 18 months**
- **18 months -2 years**
- **2 - 3 years**
- **3 - 4 years**
- **4 - 5 years**
- **5+ years**

a. What was handled well over this time?

....<br>..<br>..<br>..<br>..<br>..<br>..<br>...

**b. How could your experience have been improved?**
....<br>..<br>..<br>..<br>..<br>..<br>..<br>...

**13. Were there any assessments (e.g. neuroimaging, blood tests, cognitive tests) that you felt uncomfortable with?**
....<br>..<br>..<br>..<br>..<br>..<br>..<br>...

**a. If so, how could these have been improved?**
....<br>..<br>..

………
………
………
………
………………………………………………………………………………………

14. How did you feel after each consultation with your dementia specialist(s)?

………
………
………
………
………
………
………
………………………………………………………………………………………

15. Did you feel any questions you had were addressed during the assessment and diagnosis?
(Please circle your response) Yes No

a. If yes, what helped you to feel this way?

………
………
………
………
………
………
………
………………………………………………………………………………………

b. If no, how could the experience have been improved?

....
....
....
....
....
....
....
....

Section 3. The Diagnosis Process

16. How was the information about the diagnosis delivered to you?

....
....
....
....
....
....
....
....

a. How did the process make you feel?

....
....
....
....

……… ……… ……… ………………………………………………………………………………………………

**17. How well did the dementia specialist(s) help you understand the diagnosis?**
……… ……… ……… ……… ……… ……… ……… ……… ……………………………………………………………………………………………<br><br>a. Could this have been improved? (i.e. was there anything that the specialists could have said or done to better support/inform or reassure you)____<br>……… ……… ……… ……… ……… ……… ……… ………

**18. Did the dementia specialist(s) use any medical terms that you felt:**	
**Were really well explained? Yes/No If yes, please state these.**	**b. Were poorly explain? Yes/No – If yes, please state these.**

19. How could your overall experience of receiving a diagnosis have been improved?
....

Thank you very much for completing the first round of our Delphi study on improving the diagnosis of young onset dementia. We will now spend some time analysing your responses and will create a new questionnaire based on all the responses we receive.

When you are ready, if you could return the two questionnaires in the pre-paid envelope that would be much appreciated.

Many thanks again and we will be in touch again soon.

The Angela Project: Delphi-EXPERIENCE Questionnaire

Family Members/Supporters version

Thank you very much for agreeing to act as a Delphi Expert panel member for our study which aims to improve the diagnostic process for people living with young onset dementia and their family members/supporters. The study will consist of a minimum of three rounds. This is round one.

We would welcome your views as a family member/supporter to someone with a diagnosis of young onset dementia. In this questionnaire we hope to learn more about your experiences during the dementia diagnosis period. Our goal is to understand what worked well in the service(s) your relative/friend received during the diagnostic process and what could be improved.

What will I have to do?

There will be a series of questions about the referral, assessment and diagnostic processes of your relative's/friend's dementia diagnosis. Please answer and reflect on your own experiences. You can write as little or as much as you wish.

Please respond to all questions as this means that we can compare results in a consistent way. This should take approximately 30-45 minutes to complete depending on how detailed your responses are.

Thank you for your valuable assistance in completing this questionnaire.
If you would like any support in completing the questionnaire, please contact the researcher before starting the questionnaire and she will provide you with the relevant support.

Please insert the participant code number the researcher gave you: ....

Initial questions:

1. What is your relation to the person who received the diagnosis of young onset dementia?

   ....

2. What is your current age?

   ....

3. How old was your relative/friend when they received their diagnosis?

   ....

4. When did your relative/friend receive their diagnosis?

   ....

5. Please state the official diagnosis they were given:
   ....

6. Before your relative/friend was given a diagnosis of dementia, were they given other diagnoses as a possible reason for their symptoms? (Please circle) Yes No

   If yes, please state these below:

   ....

7. In which town or county of the UK do you live?
   ....

Section 1. The Referral Process

8. Please tell us about the experience of the referral from your GP:

....
....
....
....
....
....
....
....
....

c. Which aspects were handled well?

....
....
....
....
....
....
....
....

d. How could your experience could have been improved?

....
....
....
....
....
....
....
....

9. How did the health professionals communicate between each other during your relative's/friend's assessment/diagnosis?

……
……
……
……a
. Did this have any effect on you?
……
……
……………………………………………………………………………………………………

10. How might the various appointments with dementia specialists have been better managed to suit you? (i.e. location, time of appointment, form of contact).

……
……
……
……
……
……
……
……………………………………………………………………………………………

Section 2. The Dementia Assessment Process

**11. Please tell us about the assessment your relative/friend received from a dementia specialist(s)?**
……………………………………………………………………………………………………<br>……………………………………………………………………………………………………<br>……………………………………………………………………………………………………<br>……………………………………………………………………………………………………<br>……………………………………………………………………………………………………<br>……………………………………………………………………………………………………<br>……………………………………………………………………………………………………<br>……………………………………………………………………………………

**b. Can you comment on what was helpful?**
……………………………………………………………………………………………………<br>……………………………………………………………………………………………………<br>……………………………………………………………………………………………………<br>……………………………………………………………………………………………………<br>……………………………………………………………………………………………………<br>……………………………………………………………………………………………………<br>……………………………………………………………………………………………………<br>……………………………………………………………………………………

**12. Based on your experience, is there anything else you would have liked to happen during this assessment process?**
……………………………………………………………………………………………………<br>……………………………………………………………………………………………………<br>……………………………………………………………………………………………………

…… …… …… …… ………………………………………………………………………………………………………

**13. How helpful were the staff you met during the assessment process?**
…… …… …… …… …… …… …… ………………………………………………………………………………………………………

**14. How long did it take from being referred onto a dementia specialist by the GP to receiving the diagnosis of dementia? Please circle the corresponding time-frame:**
• **0-6 months**<br>• **6-12 months**<br>• **12months – 18months**<br>• **18 months-2 years**<br>• **2-3 years**<br>• **3-4 years**<br>• **4-5 years**<br>• **5+ years**

**c. What was handled well over this time?**

……………………………………………………………………………………………………… ……………………………………………………………………………………………………… ……………………………………………………………………………………………………… ……………………………………………………………………………………………………… ……………………………………………………………………………………………………… ……………………………………………………………………………………………………… ……………………………………………………………………………………………………… ………………………………………………………………………………………

**d. How could the experience have been improved?**
……………………………………………………………………………………………………… ……………………………………………………………………………………………………… ……………………………………………………………………………………………………… ……………………………………………………………………………………………………… ……………………………………………………………………………………………………… ……………………………………………………………………………………………………… ……………………………………………………………………………………………………… ………………………………………………………………………………………

**15. Were there any assessments (e.g. neuroimaging, blood tests, cognitive tests) that you felt uncomfortable with?**
……………………………………………………………………………………………………… ……………………………………………………………………………………………………… ……………………………………………………………………………………………………… ……………………………………………………………………………………………………… ……………………………………………………………………………………………………… ……………………………………………………………………………………………………… ……………………………………………………………………………………………………… ………………………………………………………………………………………

**b. If so, how could these have been improved?**
……………………………………………………………………………………………………… ……………………………………………………………………………………………………… ………………………………………………………………………………………………………

……
……
……
……
…………………………………………………………………………………………………

16. How did you feel after each consultation with the dementia specialist(s)?

……
……
……
……
……
……
……
…………………………………………………………………………………………………

17. Did you feel any questions you had were addressed during the assessment and diagnosis?
(Please circle your response) Yes No

b. If yes, what helped you to feel this way?

……
……
……
……
……
……
……

……………………………………………………………………………………………………

b. If no, how could the experience have been improved?

………
………
………
………
………
………
………………………………………………………………………………………………………

Section 3. The Diagnosis Process

**18. How was the information about the diagnosis delivered to you?**
.... <br>b. How did the process make you feel? <br>...

**19. How well did the dementia specialist(s) help you understand the diagnosis?**
....

………
………
………
…………………………………………………………………………………………………

a. Could this have been improved? (i.e. was there anything that the specialists could have said or done to better support/inform or reassure you)____

………
………
………
………
………
………
………
…………………………………………………………………………………………………

20. Did the dementia specialist(s) use any medical terms that you felt:

**a. Were really well explained? Yes/No If yes, please state these.**	**b. Were poorly explained? Yes/No – If yes, please state these.**

21. How could your overall experience of receiving a diagnosis have been improved?

………
………
………
………
………
………

…… ………………………………………………………………………………………………

Thank you very much for completing the first round of our Delphi study on improving the diagnosis of young onset dementia. We will now spend some time analysing your responses and will create a new questionnaire based on all the responses we receive.

When you are ready, if you could return the two questionnaires in the pre-paid envelope that would be much appreciated.

Many thanks again and we will be in touch again soon.

Appendix 2: Delphi statements organised by diagnostic group, with their short titles and long titles.

**Diagnostic Phase**	**Short title**	**Longer title**
Referral Process	GP recognition of YOD	For the GP to identify dementia in younger people.
	Appointment notice period	Ensure there is enough notice between appointment letters being issued and the appointment.

	Convenience	Making appointments convenient for working adults.
	Being involved	Being kept in the loop and feeling involved in the assessment.
	Contact family supporters as well	Healthcare professionals should make contact with family supporters if unable to get through to the person with dementia directly regarding appointments.
	Active listening	The clinicians should listen to the person with dementia and their family as a whole.
	Single point of contact	Having an identified key person as a single point of contact throughout the whole diagnostic process.
	Meeting in person	Communication with clinicians should ideally be in person.
	Avoid repetition	Avoid the same questions being asked by the separate clinicians where possible.
Assessment Process	Quick Referral	The referral process from GP to first assessment needs to be shorter.
	Referrals to specialist services	Referrals should ideally be made to specialist YOD clinicians and services.
	Considerate use of language	Clinicians should be compassionate, empathic and respectful during the assessment and particularly sensitive when providing information about a diagnosis.
	Home visits	To be seen at home for assessments and post-diagnostic support where appropriate.
	Time to ask questions	Giving the person with dementia and their family enough opportunities to ask questions.
	Calm approach	Clinicians should be calm, approachable and easy to talk to.
	Private discussions	Clinicians should offer opportunities for the person with dementia and their supporters to speak separately about any issues they wish to discuss.
	Multi-disciplinary team	To have a multi-disciplinary team involved in diagnosis to provide appropriate support.
	Enhanced awareness of YOD	More awareness and training on rarer dementia types as well as the issues faced by younger people with dementia in Mental Health Trusts.
	Understanding all forms of dementia	Being understanding during the assessments, especially visual tests for people with PCA.
	Private location	Assessments should be conducted in a quiet and private room.
	Explanation of assessments	Having more information on what the SPECT scanning was all about.
	Improved access to clinics	Better access to sleep and anger clinics.

	Improve MRI experience	The MRI experience should provide blankets, ear protectors to reduce noise and allow supporters to be in the room if the person wishes.
	Results issued more quickly	Results to be given in clinic more quickly.
	Shorter time to diagnosis	The time taken to achieve a formal diagnosis needs to be shortened if possible.
	Diagnosis explained	Providing the people with dementia and their families with information about their diagnosis and prognosis if they wish it.
Diagnosis Process	Using lay terms	Clinicians should explain medical terms, and what they mean in a simplified manner.
	Reaction to diagnosis	Remembering that receiving the diagnosis is a lot to take in for the person with dementia and supporter.
	Follow-up letter	Providing the person with dementia and their supporters with a letter which details the diagnosis.

Appendix 3: Evidence-based statements (short titles) in receiving a diagnosis of young onset dementia.

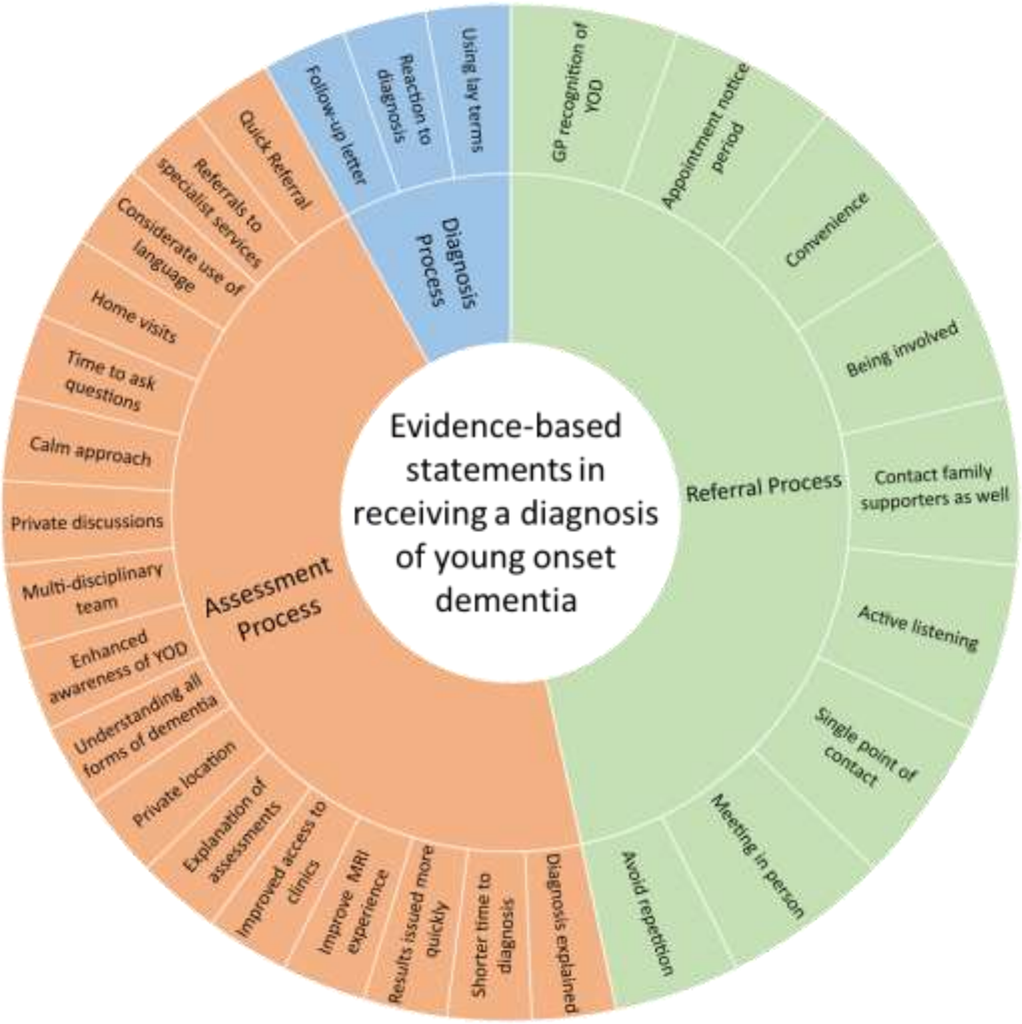
Evidence-based statements in receiving a diagnosis of young onset dementia
Referral Process
Diagnosis Process
Assessment Process
GP recognition of YOD
Appointment notice period
Convenience
Being involved
Contact family supporters as well
Active listening
Single point of contact
Meeting in person
Avoid repetition
Using lay terms
Reaction to diagnosis
Follow-up letter
Quick Referral
Referrals to specialist services
Considerate use of language
Home visits
Time to ask questions
Calm approach
Private discussions
Multi-disciplinary team
Enhanced awareness of YOD
Understanding all forms of dementia
Private location
Explanation of assessments
Improved access to clinics
Improve MRI experience
Results issued more quickly
Shorter time to diagnosis
Diagnosis explained
